# The Spectrum and Clinical Significance of Autoantibodies in Rheumatoid Arthritis

**DOI:** 10.3389/fimmu.2015.00320

**Published:** 2015-06-18

**Authors:** Geraldo da Rocha Castelar-Pinheiro, Ricardo Machado Xavier

**Affiliations:** ^1^Discipline of Rheumatology, Department of Internal Medicine, Universidade do Estado do Rio de Janeiro, Rio de Janeiro, Brazil

**Keywords:** rheumatoid factor, anti-citrullinated protein antibodies, rheumatoid arthritis, autoantibodies, genome-wide association studies

In the late 19th century, early 20th, the main known rheumatic diseases were Gout, deforming arthritis (osteoarthritis), acute rheumatism (rheumatic fever), and chronic rheumatism [rheumatoid arthritis (RA)]. There was uncertainty whether acute and chronic rheumatisms were separate diseases or a single entity with heterogeneous manifestations according to the influence of age, heredity, and environment. At the turn of the last century, the possibility of a bacterial etiology of both diseases began to be considered (1). In this scenario, the demonstration of circulating factors interacting with self-structures, later recognized as autoantibodies, contributed substantially to the establishment of the concept of autoimmunity and autoimmune diseases.

One of the earliest observations of the agglutinating capacity of rheumatoid sera was that of R. Cecil et al. at Cornell University, in 1930 (1). They found that such sera agglutinated suspensions of streptococci. H. Dawson at Columbia University, in 1932, showed that agglutination was also seen when pneumococci’s suspensions were used. These systems of agglutination were studied as diagnostic tests, but gave positive results in <50% of cases of RA (1). In 1937, while monitoring the results of Wassermann’s serology at Oslo City Hospital, Erick Waaler observed an unusual result, with agglutination rather than hemolysis of the red cells (the agglutinating activating factor) (2). In 1939, at the Third International Congress for Microbiology, in New York, Waaler presented the results on 77 RA patients, 27 of whom presented a positive test for the agglutination of erythrocytes. In 1948, using a complement fixation test for *Rickettsia*, the same phenomenon of sensitized sheep red cells agglutination with rheumatoid sera was seen and reported by H. Rose et al. at the Presbyterian Hospital (1).

The agglutinating factor was tentatively termed “rheumatoid factor (RF)” by Coggeshall et al. (3) and its identification as an antibody to antigens in the Fc region of immunoglobulin G has led to immunological studies that allowed us a better understanding of the etiopathogenesis of RA (4).

The use of latex particles instead of the sheep red cells led to a more sensitive, though less specific, assay that soon gained wide clinical acceptance (5). Automated techniques such as nephelometry and immunoturbidimetric assays gradually replaced the other semiquantitative methods because of their greater sensitivity and reproducibility, as well as adaptability to automation (6).

The introduction of enzyme-linked immunosorbent assays (ELISA) allowed a better characterization of the different isotypes of RF but their utility in clinical practice has never gained popularity. In fact, more or less at the same time precursors of what would be the anti-citrullinated peptide/protein antibody system (ACPA) were identified as a major antigenic target of RA-specific autoantibodies.

In 1964, Nienhuis and Mandema reported a novel autoantibody system designated antiperinuclear factor (APF). They showed that sera from RA patients reacted in a very specific way with keratohyalin granules present in buccal mucosa cells. Fifteen years later, also using sera from patients with RA in an indirect immunofluorescence (IFI) assay, Young et al. described the so-called “antikeratin” antibodies (AKA) on cryosections of rat esophagus. It took many years and a lot of work done by independent groups of researchers led by Hoet, Sene, Sebbag, Schellekens, and Girbauld-Neuhauser until the target of both the APF and AKA was recognized as the same antigenic protein: citrullinated (pro)filaggrin.

Despite been highly specific for RA, the difficulties in availability, standardization, and interpretation of the indirect IFI method for APF and AKA hindered the widespread popularization of these tests. The situation was drastically changed with the development of ELISA-based tests with pools of selected citrullinated peptides. Careful selection of cyclic citrullinated peptides, chosen from peptide libraries according to the ability to discriminate RA sera, was used to increase the sensitivity and specificity of the test (6, 7). Progressively, other antigenic substrates, such as citrullinated vimentin, were also shown to be useful for the clinical detection of ACPA.

Studies on the diagnostic performance of ACPA demonstrated that they are highly specific for RA and appear several years before disease onset (7). However, they are not essential for development of the RA syndrome, as a sizable number of patients (around 30–40%) do not produce them. The argument that ACPA-positive RA could be a distinct disease than ACPA-negative RA has been put forward based on clinical, epidemiological and genetic observations (7).

In terms of clinical observations, ACPA positivity has been associated with a more destructive disease course, as well as with cardiovascular complications, while ACPA negativity has been associated with drug-free remission (7).

Most interestingly, epidemiological studies demonstrated that smoking is a risk factor only for ACPA-positive RA. In addition, the RA-associated shared epitope containing alleles of the *HLA-DRB1* gene are strongly associated with the development of ACPA (8). Indeed, there is a strong interaction between these two risk factors, smoking and *HLA-DRB1* gene, demonstrating for the first time in RA a clear gene–environment interaction effect. These observations led to the proposal of a pathogenetic model in which smoke exposure induces citrullination in the lung, and *HLA-DRB1* shared epitope-restricted immune reactions to these peptides trigger systemic inflammation and, eventually, a local second hit induces chronic synovial disease. More recently, additional associations have been reported involving shared epitope *HLA-DRB1* alleles, *PTPN22* (another gene associated with RA) and smoking with the presence of specific ACPA reactivities, mainly against citrullinated α-enolase and vimentin (9).

Still in terms of genetic background, genome-wide association studies (GWAS) comparing ACPA-positive and ACPA-negative disease have shown that these two subsets of patients present different risk allele frequencies that were mainly confined to the HLA region, providing further support for distinct genetic etiologies (10). A more refined GWAS with ACPA-negative patients confirmed the HLA and several suggestive non-HLA regions of association, with a dense analysis focused on the HLA region identifying a two amino acid model (HLA-B at position 9 and HLA-DRB1 at position 11, both mapping to the peptide-binding groove of the receptor). For example, the presence of a serine at position 11 of the HLA-DRB1 would confer susceptibility to ACPA-negative, but protection to ACPA-positive disease, whereas the presence of aspartic acid or valine was a risk factor for ACPA-positive and mildly protective of ACPA-negative disease (11).

The two autoantibody systems, ACPA and RF, described for RA, have had their clinical relevacne extensively investigated in the last few years. Both have traditionally been associated with more severe disease and bone damage. In fact, a mechanism for bone damage in ACPA patients has been proposed, involving the induction and differentiation of bone resorbing osteoclasts by antibodies against citrullinated vimentin (12). In this study, ACPA titers in RA patients were correlated with serum markers for osteoclast-mediated bone resorption. The authors also identified that antibodies against mutated citrullinated vimentin bound to osteoclast surfaces and induced osteoclastogenesis and bone-resorption activity, with evidence that this effect was mediated by local release of TNF from osteoclast precursors. On the other hand, the role of RF on bone damage, independent of the presence of ACPA, is less well defined, although mechanisms involving Fc cross-linking on macrophage surface leading to pro-osteoclastogenic cytokine release have been suggested.

Some recent studies have tried to determine whether patients with isolated RF or ACPA also constitute different subsets of the disease and whether double positivity confers additional risk factor for destructive disease. Exploring potential genetic differences of these putative RA subsets, C. Terao et al. performed genotyping of the *HLA-DRB1* allele in 954 ACPA-negative Japanese patients that were negative or positive for the RF, and compared with ACPA-positive patients (13). Their conclusion was that, in fact, ACPA-negative RA includes two genetically distinctive subsets according to the presence of RF: ACPA-negative/RF-positive RA is associated with HLA-DRB1*04:05 and *09:01, whereas ACPA-negative/RF-negative RA was associated with DR14 and HLA-DR8 homozygote.

In another study, J. Sokolove et al. sought to investigate the role of RF as a contributor to the RA inflammatory burden in isolation or in synergy with ACPA (14). For that, they analyzed disease activity and serum levels of cytokines and multiple ACPA specificities in a cohort of 1,488 US veterans with RA, comparing the different groups of patients: double-negative, single-positive (ACPA or RF), and double-positive patients. They observed that the double-positive subgroup indeed had higher disease activity and higher levels of inflammatory cytokines as compared to double-negative or single-positive subgroups. In support to that, *in vitro* studies demonstrated that the presence of IgM-RF could significantly increase the production of tumor necrosis factor by macrophages stimulated with ACPA immune complexes (14).

Finally C. Hecht et al., from Erlangen, studied the effect of ACPA and RF on the number and size of bone erosion by high-resolution peripheral quantitative CT scans of the metacarpophanlangeal joints in 238 RA patients (112 double-positive, 28 RF-positive, 29 ACPA-positive, 69 double-negative) (15). They observed that double-positive patients had the highest number and size of erosions, with significant difference in the erosion number as compared to double-negative patients and in erosion size as compared to ACPA-negative patients. A linear regression mixed model showed that ACPA-positive/RF-positive status and disease duration was associated with higher number and larger size of bone erosions. In addition, the effect of RF on erosions was observed only in ACPA-positive patients. Therefore, the authors concluded that there is an interdependence of ACPA and RF in RA-mediated bone damage, being the RF a strong enhancer of the ACPA effect on the bone, and emphasizing the pathogenic role of both autoantibodies (15).

In conclusion, RF and ACPA are present in a substantial majority of RA patients, but a sizable fraction of patients is double-negative. Recent clinical and genetic studies have strongly indicated that in fact the presence of ACPA can be a marker of different subsets of RA, presenting with distinct risk factors, prognosis, and response to treatment. Additionally, the concomitant presence of both autoantibodies seems to be associated with higher disease activity and increased risk of bone damage. ACPA and RF have been included in the new 2010 ACR/EULAR Rheumatoid Arthritis Classification Criteria, demonstrating their strong discriminative diagnostic performance. With the recent data presented, it is becoming more evident that testing for these antibodies could also add important information for the therapeutic planning of these patients.

## Conflict of Interest Statement

The authors declare that the research was conducted in the absence of any commercial or financial relationships that could be construed as a potential conflict of interest.
